# Gut Hormones, Appetite Suppression and Cachexia in Patients with Pulmonary TB

**DOI:** 10.1371/journal.pone.0054564

**Published:** 2013-01-24

**Authors:** Suzanne W. Chang, William S. Pan, Daniel Lozano Beltran, Lizet Oleyda Baldelomar, Marco Antonio Solano, Iskra Tuero, Jon S. Friedland, Faustino Torrico, Robert H. Gilman

**Affiliations:** 1 Department of Medicine, George Washington University, Washington, DC, United States of America; 2 Department of International Health, Johns Hopkins Bloomberg School of Public Health, Baltimore, Maryland, United States of America; 3 CEADES (Colectivo de Estudios Aplicados y Desarollo Social) Salud y Medio Ambiente, Cochabamba, Bolivia; 4 Universidad Peruano Cayetano Heredia, Lima, Peru; 5 Department of Infectious Diseases and Immunity and The Wellcome Centre for Clinical Tropical Medicine, Imperial College London, London, United Kingdom; Institute of Infectious Diseases and Molecular Medicine, South Africa

## Abstract

**Background:**

Cachexia is a hallmark of pulmonary tuberculosis and is associated with poor prognosis. A better understanding of the mechanisms behind such weight loss could reveal targets for therapeutic intervention. The role of appetite-regulatory hormones in tuberculosis is unknown.

**Methods and Findings:**

41 subjects with newly-diagnosed pulmonary TB (cases) were compared to 82 healthy controls. We measured appetite, body mass index (BMI), % body fat (BF), plasma peptide YY (PYY), leptin, ghrelin, and resistin for all subjects. Measurements were taken at baseline for controls and at treatment days 0, 30, and 60 for cases. Baseline appetite, BMI, and BF were lower in cases than in controls and improved during treatment. PYY, ghrelin, and resistin were significantly elevated in cases and fell during treatment. Leptin was lower in cases and rose with treatment. Appetite was inversely related to PYY in cases. High pre-treatment PYY predicted reduced gains in appetite and BF. PYY was the strongest independent predictor of appetite in cases across all time points.

**Conclusions:**

Appetite-regulatory hormones are altered in TB patients. As hormones normalize during treatment, appetite is restored and nutritional status improves. High baseline PYY is an indicator of poor prognosis for improvement in appetite and nutrition during treatment. Wasting in TB patients may partly be mediated by upregulation of PYY with resulting appetite suppression.

## Introduction

Tuberculosis (TB) remains a major global public health threat, with over 1.4 million deaths reported in 2010 [1]. Cachexia is characteristic of TB with approximately two-thirds of patients presenting with dramatic weight loss [2], [3]. TB was known as consumption because of this effect. Most patients improve clinically and gain weight within weeks of starting appropriate treatment [2]. Cachexia has been linked to poor prognosis and is a major risk factor for mortality [4]–[6]. However, exact mechanisms behind this wasting are poorly understood.

Peptide YY (PYY) is a hormone secreted by the distal small intestine and large intestine that inhibits appetite through feedback into the hypothalamus. High PYY levels have been linked to decreased appetite and food intake [7]. PYY is elevated in patients with malabsorptive disorders such as inflammatory bowel disease [8], celiac sprue [9], and infectious diarrhea [10], and in patients with anorexia nervosa and cancer in the absence of known GI illness [11], [12]. Dysregulation in PYY secretion could therefore be associated with decreased food intake and subsequent weight loss in a range of disease processes. To our knowledge, there is no published data on PYY secretion in TB.

Leptin binds to hypothalamic receptors leading to decreased appetite and increased energy expenditure [13]. Produced in adipocytes, it increases with fat mass and has also been linked to inflammatory mediators, thus has emerged as a key candidate in the mechanism of infection-induced weight loss [14], [15]. Previous studies of leptin activity in TB have shown conflicting results, with authors reporting increases [16], decreases [17]–[19], or no change [20] in baseline leptin.

Resistin, also produced in adipose tissue, has been linked to insulin resistance, obesity, and type 2 diabetes in murine studies [21]. Resistin decreases food intake, possibly through blocking the orexigenic effects of neuropeptide Y (a similar pathway to PYY) [22]. Though this hormone has been studied in cancer [21], [23], [24], we found no data on resistin in infection-related cachexia.

The role of ghrelin in cachexia is unclear. Produced in endocrine cells of the stomach, ghrelin induces a positive energy balance by stimulating food intake and reducing fat utilization, acting through vagal afferent pathways to increase feeding, promote gastric emptying, decrease energy consumption, and stimulate pituitary release of growth hormone [25]. This peptide’s anti-inflammatory properties have generated substantial interest. Ghrelin decreases pro-inflammatory cytokine concentrations and muscle breakdown in inflammatory states, and peripheral injection of ghrelin protects against cytokine-mediated anorexia [25]. Circulating concentrations of the peptide are elevated in patients with cachexia resulting from cancer, COPD, and anorexia nervosa [26].

This study investigates the hypothesis that patients with newly-diagnosed TB display abnormal regulation of hormones which relate to appetite and nutritional status, and that these abnormalities trend back towards normal values as patients are treated. A better understanding of the mechanisms of appetite suppression in TB may reveal targets for therapeutic intervention to reduce cachexia and lessen the risk of mortality from this infection.

## Methods

### Study Design

This was a prospective cohort study to evaluate the effect of pulmonary TB on nutritional status, appetite, and appetite-regulatory hormone profile among infected adults living in an endemic tuberculosis region.

### Setting

The study took place in Cochabamba, the third largest city in Bolivia, with an urban population of 517,024 people [27]. Cochabamba is endemic for TB, with an incidence of smear-positive pulmonary TB of 48.6 per 100,000 people [28]. Subjects were drawn from a pool of newly-diagnosed TB patients at three health centers, which together served a catchment area of 135,410 people. The rate of multi-drug resistant TB among new cases of TB in Bolivia is estimated at only 1.2% [29].

### Subjects

The cohort subjects (‘cases’) were identified consecutively from patients with untreated pulmonary TB which had been newly-diagnosed by 2 or more sputum smear samples positive for Acid Fast Bacilli (AFB) following World Health Organization (WHO) guidelines [30]. Exclusion criteria included age less than 18 years, prior treatment for TB, and known comorbidity with diabetes mellitus (DM), HIV, malignancy, lung disease other than TB, or cardiac disease. Cases were followed during the first two months of treatment with a standard regimen of Isoniazid, Rifampin, Ethambutol, and Pyrazinamide as per the Bolivian National TB Control Program guidelines. We compared results to a control group of healthy volunteers (‘controls’) drawn from community organizations within the same geographic region as cases. Both cases and controls were screened by serology for HIV and all were negative. Baseline evaluations were performed on all subjects, including blood samples, appetite evaluation, height and weight measurements, and bioimpedance analysis. Cases had repeat evaluations at treatment days 30 and 60.

### Laboratory Evaluation

At each visit, fasting blood samples were taken for evaluation of PYY, leptin, ghrelin, and resistin. To prevent breakdown of PYY and ghrelin by proteases, tubes were prepared with aprotinin prior to blood draw according to published protocols [10].

After venipuncture, blood samples were kept on ice and centrifuged within 30 minutes. Plasma aliquots were stored at −25°C until assays were performed. PYY, leptin, and total ghrelin were assayed using Millipore ELISA kits and resistin was evaluated using the Millipore Luminex multiplex human adipokine kit as detailed by the manufacturer (www.millipore.com).

### Sputum Evaluation

According to WHO guidelines and the regulations of the Bolivian National TB Control Programme, sputum smear evaluation for AFB positivity is sufficient to diagnose a case of pulmonary TB. We therefore used this as our case definition criteria. Sputum analysis for AFB positivity was done by laboratory personnel of the three health centers included. As a quality control measure to confirm MTb infection and rule out non-tuberculous mycobacterial colonizers or contaminants in our cases we also performed cultures of the sputum specimens via the Microscopic Observation Drug Susceptibility (MODS) assay, a liquid culture medium which allows rapid detection of organisms as well as drug susceptibility testing within an average of 7 days [31]. WHO recommends use of such liquid culture media for low-income settings [32].

### Nutritional Status Evaluation

At each visit, BMI was calculated using measurements for height to the nearest 0.5 cm and weight to the nearest 0.1 kg. Bioimpedance measurements were taken using the RJL Systems Quantum II Bioelectrical Impedance Analyzer, using previously validated measurement procedures [33]. Body composition, including percent body fat (BF), was calculated using the RJL Systems Body Composition Analysis software.

### Appetite Measurement

At each visit, appetite was evaluated using a visual analog scale adaptation of the Simplified Nutritional Appetite Questionnaire [34], [35]. This scale consists of results in a score between 1 and 20, with 1 being the poorest and 20 being the best appetite. The scale has been validated for prediction of malnutrition and weight loss in outpatient populations [35].

### Statistical Evaluation

We evaluated differences in demographics between cases and controls using simple t-tests. For comparisons between cases and controls for key measures (nutritional status and hormones), we used Generalized Estimating Equations in a univariate regression to adjust for the correlated covariance structure from repeated measures among cases [36]. Thus, p-values reported are more conservative than individual comparisons for every one of the cases and controls at each follow-up time. Pearson correlations were computed for appetite, BMI, and BF versus PYY, leptin, ghrelin, and resistin for cases at each time point (baseline, days 30 and 60). Reported p-values were adjusted for multiple comparisons using Sidak’s method [37]. Due to the exploratory nature of the correlations, unadjusted p-values were also examined.

To evaluate the effects of “abnormal” hormone levels, multivariate regressions for changes in appetite, BF, and BMI during treatment were fit for extreme pre-treatment values of each hormone. Values in cases were categorized as above, below or within the 95% confidence interval of control values. These categories were then regressed on the amount of change observed in nutritional status, controlling for baseline nutritional status and using “within the 95% confidence interval of control values” as the reference group. For example, if the outcome was change in appetite from baseline to day 30, two predictors would be a 3-level categorical variable for PYY and the baseline appetite score. Only appetite changes from baseline to day 30 and BF and BMI changes from baseline to day 60 were included, as changes during other treatment intervals were not significant (Table 1).

**Table 1:** Appetite, Nutritional Status, and Hormones Over Time in Patients Receiving Treatment for Pulmonary TB.

Variables	Controls	Cases			Comparisons (*p*-values)		
	(n = 82)	Day 1 (n = 41)	Day 30 (n = 35)	Day 60 (n = 35)	Controls vs Day 1	Controls vs Day 30	Controls vs Day 60
**Age**	32 (29,34)	34 (29,38)	–	–	0.428	–	–
**Sex** (#M/#F)	43/39	24/17	–	–	0.561	–	–
**Appetite^1^**	14.6 (14.1, 15.1)	12 (11.4, 12.8)	14.2 (13.3, 15.1)	14.7 (14.1, 15.6)	**<0.001**	0.34	0.64
**BMI**	24.3 (23.5, 25.2)	20.7 (19.5, 21.9)	21.2 (20.0, 22.3)	21.7 (20.5, 22.9)	**<0.001**	**<0.001**	**<0.001**
**% Body Fat**	28.1 (26.4, 29.8)	21.8 (19.4, 24.1)	22.6 (20.1, 25.0)	24.6 (22.2, 27.0)	**<0.001**	**<0.001**	**0.021**
**Peptide YY^2^**	81.0 (71.5, 90.5)	164.6 (129, 200)	90.9 (66.8, 115)	78.1 (60.8, 95.4)	**<0.001**	0.46	0.77
**Leptin^2^**	9.9 (8.0, 11.9)	3.2 (1.8, 4.7)	5.0 (2.9, 7.1)	6.4 (3.6, 9.1)	**<0.001**	**<0.001**	**0.04**
**Ghrelin^2^**	1166 (1110, 1223)	1579 (1367, 1791)	1237 (1070, 1403)	1185 (1028, 1341)	**0.002**	0.43	0.83
**Resistin^2^**	18466 (16675, 20297)	26843 (29583, 44104)	22339 (17889, 26790)	12642 (5136, 20148)	**<0.001**	0.12	0.14

* Results are reported as means (95% confidence interval).

^1^Appetite was measured using a quantitative score ranging from 1–20; see methods section for further details.

^2^Hormone levels are reported as fasting plasma levels in pcg/mL.

## Results

Between January and June of 2009, 41 cases and 82 controls were evaluated. There was no significant difference in age or sex between the case and control groups (Table 1). Of the 41 cases, Mycobacterium tuberculosis was isolated by MODS in 38 instances. INH mono-resistance was detected in 2 of these, and the remaining cases were pan-sensitive. Three specimens failed to grow in MODS. As these three cases were smear-positive, we believe this represented laboratory complications rather than true negatives. However, to ensure this did not alter our results we ran the below analyses both with and without those three cases, and there were no significant differences in main outcomes. Of the 41 cases, four were lost to follow-up and two developed drug reactions and were excluded from the study after the first visit. Thirty-five cases are therefore included in our complete analysis. Per Bolivian National TB Control Program guidelines, all included cases underwent repeat sputum smear analysis after 60 days of treatment and were AFB negative.

### Appetite and Nutritional Status

Baseline appetite was lower in cases than in controls (p<0.001) and improved rapidly during treatment. Mean appetite scale in cases rose 17% from baseline to day 30, at which point mean appetite of cases reached that of the control group. Similarly, baseline BMI and BF were lower in cases than in controls (p<0.001) and improved during treatment, though remained lower than controls by treatment day 60 (Table 1).

### Appetite-Regulatory Hormones

**Figure 1:**
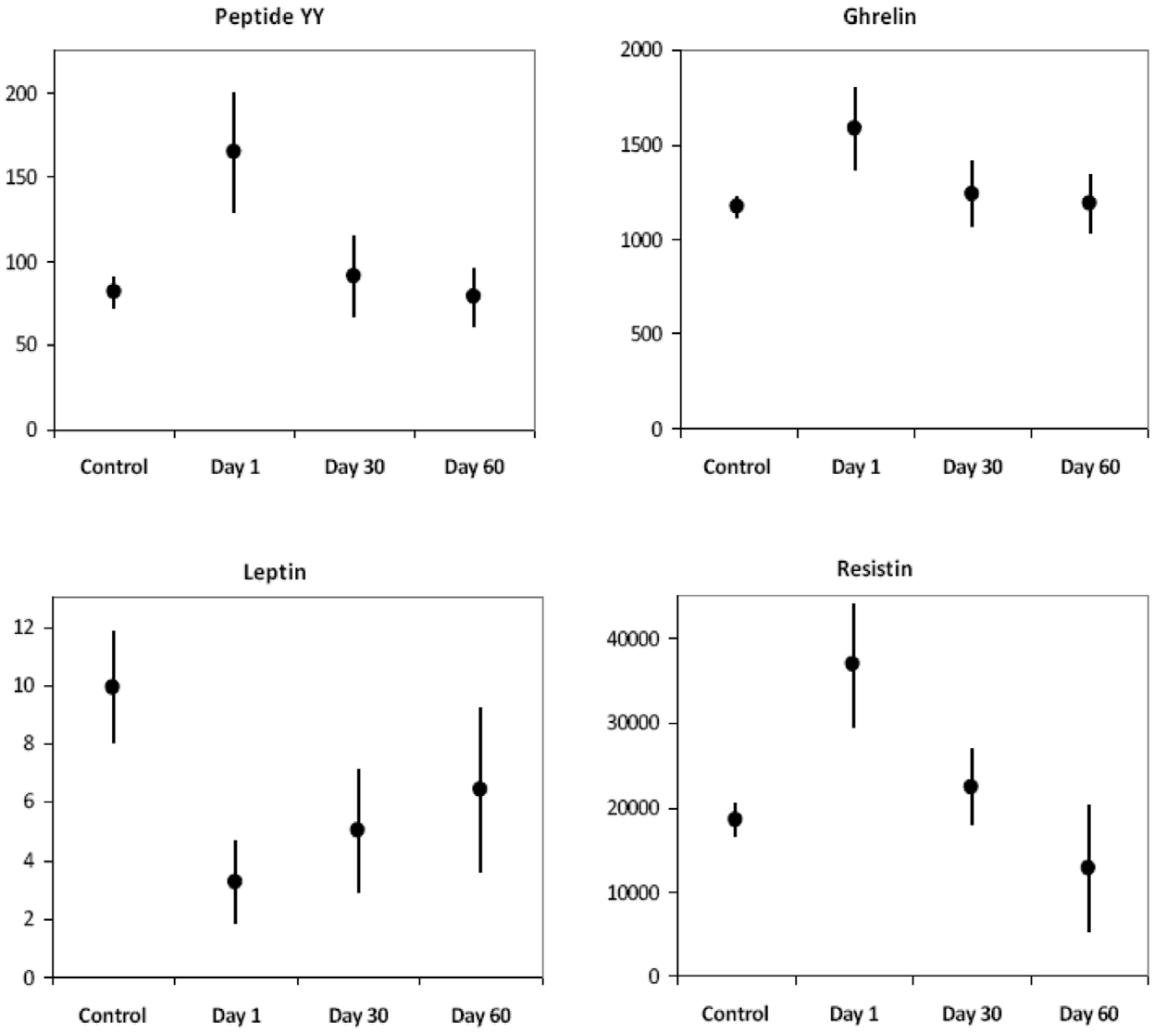
PYY, Ghrelin, and Leptin in Patients Receiving Treatment for TB and in Healthy Controls. Dots represent mean values. Bars represent 95% confidence intervals. The Y axis is pcg/ml.

Mean PYY was elevated at 164.6 pcg/ml in pre-treatment cases, approximately twice the plasma concentration of controls (Figure 1, Table 1). PYY concentrations decreased 45% from baseline during the first 30 days of treatment (p<0.0001) at which time they were not significantly different from control values. There was a non-significant 14% PYY decline from day 30 to 60 (Table 1, Figure 1).

Baseline leptin was three-fold lower in cases than in controls (3.2 pcg/ml vs 9.9 pcg/ml, p<0.001) and increased significantly during each treatment interval (p = 0.004 baseline to day 30, p = 0.036 day 30 to 60). Even by day 60, leptin levels remained below control values (p = 0.035). Ghrelin was 35% higher at baseline in cases compared with controls (1579 pcg/ml vs 1166 pcg/ml, p = 0.002) and declined with treatment, decreasing 22% by treatment day 30 (p<0.0001). Baseline resistin in cases was approximately twice that of controls (36843 pcg/ml vs 18486 pcg/ml, p<0.001), and declined approximately 40% during each treatment interval (Table 1, Figure 1).

### Predictors of Appetite

**Figure 2:**
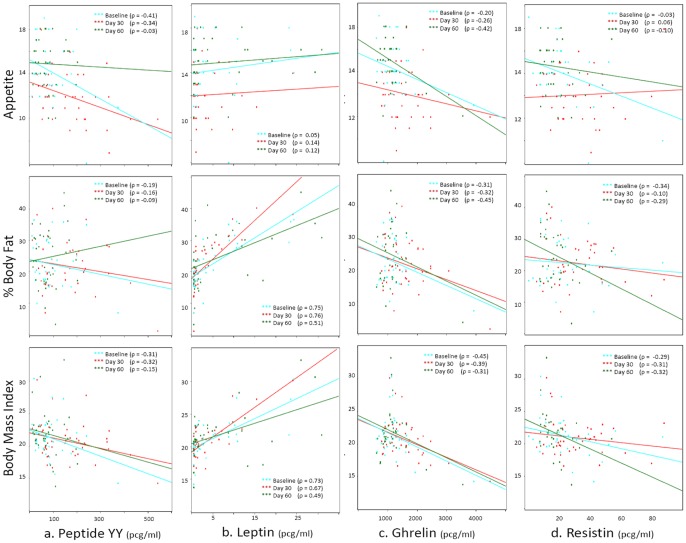
Correlations between Hormones, Appetite, and Nutritional Status in Patients with Pulmonary TB.

In univariate linear regression analysis examining all lab variables, PYY was the strongest predictor of appetite in cases. No other hormones were significant predictors across multiple time points. PYY was a marker of poor prognosis for appetite gain during treatment, with abnormally high levels of PYY predicting a significantly smaller appetite gain than a PYY within the normal range (1.6 unit increase vs 3.7 unit increase, p = .06). No other variables achieved a p-value under 0.2 to explain changes in appetite. Among cases, appetite was negatively correlated with PYY across all time points, which indicates that a decline in PYY corresponded to an increase in appetite (Figure 2a). The correlation remained a strong trend but no longer reached significance when p-values were adjusted for multiple comparisons (p = 0.07, 0.29, and 1.0 for treatment days 0, 30, and 60 respectively).

### Predictors of Nutritional Status

High PYY was an indicator of poor prognosis for BF gain during treatment, with baseline PYY above the normal range predicting significantly smaller increase in BF compared to low PYY values (0.6% vs. 7.3%, p = 0.004). Among cases, BMI and BF rose with increasing leptin concentrations at all time points (Figure 2b). BMI in cases was inversely correlated with PYY (Figure 2a), ghrelin (Figure 2c), and resistin (Figure 2d) across all time points, though these did not reach significance when p-values were corrected for multiple comparisons.

## Discussion

Cachexia is a common finding in pulmonary TB and is linked to poor prognosis. The purpose of this study was to better understand the hormonal mediators of appetite and nutrition in patients undergoing treatment for pulmonary TB.

In this first published study examining PYY in pulmonary TB, the key finding was that a high pre-treatment PYY was an indicator of poor prognosis for gains in both appetite and BF during treatment. PYY was the strongest independent predictor of poor appetite in cases. Higher PYY concentrations corresponded to lower BMI and appetite in cases at multiple time points, again supporting a link between high PYY and poor nutritional outcomes. PYY appears to play a key role in appetite regulation and resulting nutritional status changes in patients undergoing treatment for TB.

We found marked elevations in PYY, ghrelin, and resistin and reductions in plasma leptin in cases compared to healthy controls. During TB treatment, these abnormal hormone concentrations normalized rapidly, with only leptin remaining significantly decreased by day 30. Our results also revealed differences in mediators of appetite and nutritional status between cases and controls. In cases, PYY was the strongest negative predictor of appetite and leptin did not have a significant effect. In controls, appetite had a weakly positive correlation with PYY and negative correlation with leptin. These key differences show that normal physiology is disrupted in infection, suggesting that not only increased energy expenditure, but also abnormal control of appetite and resulting anorexia contribute to wasting in TB.

We found that alterations in energy regulatory hormones correlate with changes in appetite and body composition in patients undergoing treatment. Appetite, BMI, and BF were all decreased in cases compared to controls, and rose during treatment as would be predicted, though BMI and BF lagged behind appetite and had not improved to control levels by 60 days into treatment. One probable explanation for this is that appetite recovers first, and markers of nutritional status are slower indicators of improvement as TB is treated.

We know of no previous studies of PYY in TB. However, our results are consistent with previous work from our group in diarrheal disease as well as other studies demonstrating negative correlations between PYY and appetite [38], [39]. Our findings also support the results of Moschovi et al, who demonstrated high PYY levels in acute leukemia with associated weight loss and found that PYY trended down with treatment and was inversely related to BMI [40]. We propose that abnormal PYY elevations in TB disease result in appetite suppression, which helps drive the wasting process.

We found that leptin concentrations were decreased in TB patients and rose with treatment, were unrelated to cytokines but were strongly related to BF/BMI. This correlation between leptin and body mass confirms results of multiple prior studies [18]–[20], [41] and is expected since leptin is produced in adipose tissue. These findings, combined with a lack of significant correlation between leptin and appetite, suggest that leptin reductions in TB are a reflection of wasting seen in TB disease, rather than a driving force behind appetite and nutritional dysregulation.

We found that ghrelin in TB patients is elevated compared to controls, falls with treatment, and correlates negatively with BMI and BF. Our findings conflict with the one prior study we found on ghrelin levels in TB, which reported no differences in baseline or post-treatment ghrelin concentrations in TB patients and reported lower ghrelin levels in malnourished cases compared to well-nourished cases [20]. Our results do agree with studies examining ghrelin in other pulmonary disorders, which found elevated ghrelin in malnourished patients with COPD and lung cancer [42], [43].

While no other published studies have examined resistin in infections, our finding of elevated resistin in the disease state agreed with prior studies showing elevations in gastrointestinal cancers [23], [44], direct correlations between resistin and cancer stage [23] and resistin and BMI loss [21].

In summary, our data show that patients with pulmonary TB display clear alterations in energy regulatory hormones in comparison to healthy controls, and these alterations coincide with changes in appetite and nutritional status. As altered hormone levels normalized during treatment, appetite and nutritional status also improved. PYY was the strongest predictor of appetite in these patients and high PYY was an indicator of poor prognosis, with high levels predicting reduced gains in appetite and body fat during treatment. While previous studies have examined various combinations of energy-regulatory hormones in patients with TB, we are unaware of any studies which have evaluated PYY, leptin, ghrelin, and resistin in the same population, or any that have three longitudinal data points during treatment. This broad view provides valuable insight into the patterns of disrupted energy regulation and inflammation in TB. In addition, this was the first published study to examine PYY in TB and our results suggest this hormone is a key player in appetite and energy dysregulation in TB.

### Implications for Future Research

Alterations in PYY secretion may be an important mechanism regulating appetite loss and wasting in TB. Future development of PYY inhibitors or receptor antagonists may be beneficial in combating appetite suppression in TB, with a goal of increasing food intake and reducing wasting. Modulating PYY activity is already being investigated as a treatment for obesity [7], [45]. Finally, we have shown a range of abnormalities in easily-measured gut hormones associated with appetite and weight loss which deserve investigation as potential biomarkers of treatment response in TB patients.

### Limitations

The relatively short follow-up time of this study limited our ability to measure long-term correlations between hormones, appetite, and nutritional status during treatment. While we found strong correlation trends between PYY and appetite as well as BF, we did not detect a correlation between PYY and BMI gain, nor could we detect correlations between appetite and BMI/BF gain during treatment. BMI and BF likely lag behind appetite, with appetite improving first during treatment and weight gain happening as a result. Thus, a longer follow-up time may have demonstrated stronger correlations between initial PYY and appetite and weight changes during or following treatment.

To rule out the possibility that changes in hormones reflect differences in body composition rather than the disease state itself, it would have been ideal to match cases and controls by BMI and BF. However, as TB generally causes cachexia, healthy subjects by nature do not have equivalent body composition to TB patients and thus BMI was not a feasible option to use as matching criteria. A future study comparing TB patients with those with other cachexia-inducing disease states could further explore the hormonal abnormalities specific to TB.

## References

[1] World Health Organization (2010) Global Tuberculosis Control: A Short Update to the 2009 Report. Geneva, Switzerland: WHO.

[2] Eddleston M, Davidson R, Brent A, Wilkinson R (2008) Oxford Handbook of Tropical Medicine, 3rd Edition. Oxford, UK: Oxford University Press. 856 p.

[3] Fauci AS, Braunwald E, Kasper DL, Hauser SL (2009) Harrison’s Principles of Internal Medicine, 17th Edition. New York: McGraw-Hill Professional. 2958 p.

[4] Sacks LV, Pendle S (1998) Factors related to in-hospital deaths in patients with tuberculosis. Arch Intern Med 158: 1916–1922. 9759688 10.1001/archinte.158.17.1916

[5] Lubart E, Lidgi M, Leibovitz A, Rabinovitz C, Segal R (2007) Mortality of patients hospitalized for active tuberculosis in Israel. Isr Med Assoc J 9: 870–873. 18210928

[6] Santo AH, Pinheiro CE, Jordani MS (2003) [Multiple-causes-of-death related to tuberculosis in the State of Sao Paulo, Brazil, 1998]. Rev Saude Publica 37: 714–721. 14666300 10.1590/s0034-89102003000600005

[7] Vincent RP, le Roux CW (2008) The satiety hormone peptide YY as a regulator of appetite. J Clin Pathol 61: 548–552. 18441153 10.1136/jcp.2007.048488

[8] Adrian TE, Savage AP, Bacarese-Hamilton AJ, Wolfe K, Besterman HS, et al (1986) Peptide YY abnormalities in gastrointestinal diseases. Gastroenterology 90: 379–384. 3753594 10.1016/0016-5085(86)90936-4

[9] Wahab PJ, Hopman WP, Jansen JB (2001) Basal and fat-stimulated plasma peptide YY levels in celiac disease. Dig Dis Sci 46: 2504–2509. 11713961 10.1023/a:1012344424300

[10] Beck AL, Cabrera L, Pan WK, Cama V, Friedland JS, et al (2008) Peptide YY: a gut hormone associated with anorexia during infectious diarrhea in children. J Pediatr 153: 677–682. 18571670 10.1016/j.jpeds.2008.04.065 PMC5498098

[11] Misra M, Miller KK, Tsai P, Gallagher K, Lin A, et al (2006) Elevated peptide YY levels in adolescent girls with anorexia nervosa. J Clin Endocrinol Metab 91: 1027–1033. 16278259 10.1210/jc.2005-1878

[12] Nakahara T, Kojima S, Tanaka M, Yasuhara D, Harada T, et al (2007) Incomplete restoration of the secretion of ghrelin and PYY compared to insulin after food ingestion following weight gain in anorexia nervosa. J Psychiatr Res 41: 814–820. 17054989 10.1016/j.jpsychires.2006.07.021

[13] Ukkola O (2004) Peripheral regulation of food intake: new insights. J Endocrinol Invest 27: 96–98. 15053251 10.1007/BF03350918

[14] Faggioni R, Fantuzzi G, Fuller J, Dinarello CA, Feingold KR, et al (1998) IL-1 beta mediates leptin induction during inflammation. Am J Physiol 274: R204–208. 9458919 10.1152/ajpregu.1998.274.1.R204

[15] Faggioni R, Feingold KR, Grunfeld C (2001) Leptin regulation of the immune response and the immunodeficiency of malnutrition. FASEB J 15: 2565–2571. 11726531 10.1096/fj.01-0431rev

[16] Cakir B, Yonem A, Guler S, Odabasi E, Demirbas B, et al (1999) Relation of leptin and tumor necrosis factor alpha to body weight changes in patients with pulmonary tuberculosis. Horm Res 52: 279–283. 10965207 10.1159/000023495

[17] Buyukoglan H, Gulmez I, Kelestimur F, Kart L, Oymak FS, et al (2007) Leptin levels in various manifestations of pulmonary tuberculosis. Mediators Inflamm 2007: 64859. 17497033 10.1155/2007/64859 PMC1804295

[18] van Crevel R, Karyadi E, Netea MG, Verhoef H, Nelwan RH, et al (2002) Decreased plasma leptin concentrations in tuberculosis patients are associated with wasting and inflammation. J Clin Endocrinol Metab 87: 758–763. 11836317 10.1210/jcem.87.2.8228

[19] Schwenk A, Hodgson L, Rayner CFJ, Griffin G, Macallan DC (2003) Leptin and energy metabolism in pulmonary tuberculosis. American Journal of Clinical Nutrition 77: 392–398. 12540399 10.1093/ajcn/77.2.392

[20] Kim JH, Lee CT, Yoon HI, Song J, Shin WG, et al (2010) Relation of ghrelin, leptin and inflammatory markers to nutritional status in active pulmonary tuberculosis. Clin Nutr 29(4): 512–518. 20153570 10.1016/j.clnu.2010.01.008

[21] Kerem M, Ferahkose Z, Yilmaz UT, Pasaoglu H, Ofluoglu E, et al (2008) Adipokines and ghrelin in gastric cancer cachexia. World J Gastroenterol 14: 3633–3641. 18595130 10.3748/wjg.14.3633 PMC2719226

[22] Cifani C, Durocher Y, Pathak A, Penicaud L, Smih F, et al (2009) Possible common central pathway for resistin and insulin in regulating food intake. Acta Physiol (Oxf) 196: 395–400. 19183337 10.1111/j.1748-1716.2008.01949.x

[23] Nakajima TE, Yamada Y, Hamano T, Furuta K, Gotoda T, et al (2009) Adipocytokine levels in gastric cancer patients: resistin and visfatin as biomarkers of gastric cancer. J Gastroenterol 44: 685–690. 19430715 10.1007/s00535-009-0063-5

[24] Nakajima TE, Yamada Y, Hamano T, Furuta K, Oda I, et al (2010) Adipocytokines and squamous cell carcinoma of the esophagus. J Cancer Res Clin Oncol 136: 261–266. 19693538 10.1007/s00432-009-0657-6 PMC11827938

[25] Asakawa A, Inui A, Kaga T, Yuzuriha H, Nagata T, et al (2001) Ghrelin is an appetite-stimulatory signal from stomach with structural resemblance to motilin. Gastroenterology 120: 337–345. 11159873 10.1053/gast.2001.22158

[26] Kamiji MM, Inui A (2008) The role of ghrelin and ghrelin analogues in wasting disease. Curr Opin Clin Nutr Metab Care 11: 443–451. 18542005 10.1097/MCO.0b013e328303dee4

[27] Instituto Nacional de Estadistica de Bolivia (2001) Censo de Poblacion y Vivienda 2001.

[28] Programa Nacional de Control de Tuberculosis (2008) Bolivia.

[29] World Health Organization (2010) Multidrug and extensively drug-resistant TB (M/XDR-TB); 2010 Global Report on Surveillance and Response. Geneva, Switzerland: WHO.

[30] World Health Organization (2010) Treatment of Tuberculosis: Guidelines. Geneva, Switzerland: WHO. 23741786

[31] Moore DA, Evans CA, Gilman RH, Caviedes L, Coronel J, et al (2006) Microscopic-observation drug-susceptibility assay for the diagnosis of TB. N Engl J Med 355: 1539–1550. 17035648 10.1056/NEJMoa055524 PMC1780278

[32] World Health Organization (2007) Use of Liquid TB Culture and Drug Susceptibility Testing (DST) in Low and Medium Income Settings. Geneva, Switzerland: WHO.

[33] (2007) Quantum II and Quantum X Bioelectrical Impedance Analyzers.

[34] Wilson MM, Thomas DR, Rubenstein LZ, Chibnall JT, Anderson S, et al (2005) Appetite assessment: simple appetite questionnaire predicts weight loss in community-dwelling adults and nursing home residents. Am J Clin Nutr 82: 1074–1081. 16280441 10.1093/ajcn/82.5.1074

[35] Neelemaat F, Kruizenga HM, de Vet HC, Seidell JC, Butterman M, et al (2008) Screening malnutrition in hospital outpatients. Can the SNAQ malnutrition screening tool also be applied to this population? Clin Nutr 27: 439–446. 18395946 10.1016/j.clnu.2008.02.002

[36] Zeger SL, Liang K-Y (1986) Longitudinal data analysis for discrete and continuous outcomes. Biometrics 42: 121–130. 3719049

[37] Sidak Z (1967) Rectangular confidence regions for the means of multivariate normal distribution. Journal of the American Statistical Association 62: 626–633.

[38] Chaudhri O, Small C, Bloom S (2006) Gastrointestinal hormones regulating appetite. Philos Trans R Soc Lond B Biol Sci 361: 1187–1209. 16815798 10.1098/rstb.2006.1856 PMC1642697

[39] Chaudhri OB, Salem V, Murphy KG, Bloom SR (2008) Gastrointestinal satiety signals. Annu Rev Physiol 70: 239–255. 17937600 10.1146/annurev.physiol.70.113006.100506

[40] Moschovi M, Trimis G, Vounatsou M, Katsibardi K, Margeli A, et al (2008) Serial plasma concentrations of PYY and ghrelin during chemotherapy in children with acute lymphoblastic leukemia. J Pediatr Hematol Oncol 30: 733–737. 19011469 10.1097/MPH.0b013e318179a1d8

[41] Santucci N, D’Attilio L, Kovalevski L, Bozza V, Besedovsky H, et al (2011) A multifaceted analysis of immune-endocrine-metabolic alterations in patients with pulmonary tuberculosis. PLOS One 6: e26363. 22022605 10.1371/journal.pone.0026363 PMC3192801

[42] Itoh T, Nagaya N, Yoshikawa M, Fukuoka A, Takenaka H, et al (2004) Elevated plasma ghrelin level in underweight patients with chronic obstructive pulmonary disease. Am J Respir Crit Care Med 170: 879–882. 15271696 10.1164/rccm.200310-1404OC

[43] Shimizu Y, Nagaya N, Isobe T, Imazu M, Okumura H, et al (2003) Increased plasma ghrelin level in lung cancer cachexia. Clin Cancer Res 9: 774–778. 12576449

[44] Nakajima TE, Yamada Y, Hamano T, Furuta K, Matsuda T, et al (2010) Adipocytokines as new promising markers of colorectal tumors: adiponectin for colorectal adenoma, and resistin and visfatin for colorectal cancer. Cancer Sci 101: 1286–1291. 20331631 10.1111/j.1349-7006.2010.01518.x PMC11159666

[45] Renshaw D, Batterham RL (2005) Peptide YY: a potential therapy for obesity. Curr Drug Targets 6: 171–179. 15777187 10.2174/1389450053174523

